# Evolution of genome base composition and genome size in bacteria

**DOI:** 10.3389/fmicb.2012.00420

**Published:** 2012-12-06

**Authors:** Hiromi Nishida

**Affiliations:** Agricultural Bioinformatics Research Unit, Graduate School of Agricultural and Life Sciences, University of TokyoTokyo, Japan

In bacteria and archaea, genome size and guanine–cytosine (GC) content are correlated (Bentley and Parkhill, 2004; Musto et al., 2006; Mitchell, 2007; Suzuki et al., 2008; Guo et al., 2009). These parameters show greater correlation in bacteria (Pearson's correlation coefficient *r* = 0.46) than in archaea (*r* = 0.195) (Nishida, 2012a). The GC content in bacteria varies widely from 13.5% in “*Candidatus* Zinderia insecticola” (McCutcheon and Moran, 2010) to 74.9% in *Anaeromyxobacter dehalogenans* (Thomas et al., 2008). Although the GC content is similar among closely related bacteria, sometimes, the GC content is similar in phylogenetically distant bacteria. The distribution of GC content in bacterial genomes differs from a Gaussian distribution with multiple peaks.

Bacterial chromosome organization is mediated by nucleoid-associated proteins (NAPs) (Wang et al., 2011). The specificity of NAP–DNA binding is determined by the differences in the GC content in specific regions of the DNA (Lucchini et al., 2006; Navarre et al., 2006; Castang et al., 2008; Smits and Grossman, 2010; Yun et al., 2010; Gordon et al., 2011). For example, the *Salmonella* NAP specifically binds to DNA regions with low GC content and inhibits expression of the genes present in these regions (Lucchini et al., 2006; Navarre et al., 2006). NAPs vary among bacteria (Ali et al., 2012). In addition, the NAP genes are located in the plasmid as well as in the chromosome, suggesting that these genes have been distributed via plasmids (Takeda et al., 2011). I hypothesize that the GC content distribution may be related to the variation in bacterial NAPs (Nishida, 2012b, 2013). However, the correlation between the genome size and GC content in bacteria is poorly understood.

The obligate host-associated bacteria contain short genomes with low GC content (Mira et al., 2002; Moran, 2002; McCutcheon and Moran, 2012). Insertion sequence elements play an important role in the genome reduction of the host-associated bacteria (Song et al., 2010), whose small population size and asexual mode of reproduction lead to reduction of the genome size. In addition, deletion of the genes involved in DNA repair may contribute to a GC-poor genome (Moran et al., 2008). However, genome size reduction is not limited to the obligate host-associated bacteria (Nilsson et al., 2005). Generally, bacteria show a bias toward genomic deletions than insertions (Mira et al., 2001). Thus, bacteria must acquire additional genes to adapt to different environments.

Some bacterial lineages, for example, Actinobacteria, have maintained long genomes with high GC content. Plasmids (and viruses) have played an important role in additional gene uptake into chromosomes (Davison, 1999; Sørensen et al., 2005; Harrison and Brockhurst, 2012). Occasionally, the plasmid DNA gets integrated into the host chromosomal DNA (Harrison and Brockhurst, 2012). In addition, viral DNA occasionally remains in the chromosome as a prophage. Horizontally transferred DNA, plasmid DNA, and virus DNA have lower GC content than host chromosome DNAs (Rocha and Danchin, 2002). In a previous study, I compared the GC content across 953 pairs of bacterial chromosomes and plasmids. Among the 953 pairs, 746 (78.3%) pairs showed <10% difference in the GC content of the plasmid DNA and the host chromosomal DNA (Nishida, 2012a). Probably, most bacteria are unable to maintain and regulate plasmids that show very different GC content from their own. However, why most bacteria have not acquired DNAs with GC content higher than that of their own chromosome, but have acquired DNAs with lower GC content is not clear.

During evolution, DNA base mutations occurred intracellulaly and not in extracellular environments. The DNA polymerase components that are involved in DNA replication directly influence the base composition of the genome (Zhao et al., 2007; Wu et al., 2012). Variations in the bacterial genome DNA sequences cannot be fully explained on the basis of neutral mutations alone (Sueoka, 1988). In bacterial genomes, mutations from GC to adenosine–thymine (AT) are more common than mutations from AT to GC (Lind and Andersson, 2008; Hershberg and Petrov, 2010; Hildebrand et al., 2010; Rocha and Feil, 2010). I hypothesize that plasmids (and viruses) have been generated from the chromosome (Frontiers Research Topics, “Evolution and function of bacterial and archaeal genome sequences”). The host bacterial genome DNA has undergone a series of changes during evolution to become AT rich. On the other hand, the GC content in a plasmid that is in an extracellular environment would not change. Such a plasmid will not be accepted by the original host bacterium because most bacteria appear unable to acquire DNAs with GC content higher than that in their own chromosome. Such plasmids are transferred to bacteria that contain genomes with GC content higher than that in the plasmid (Figure 1). This natural system may be effective for obtaining useful genetic information (DNA fragments) from phylogenetically distant bacteria. I propose that the genome size and GC content in bacteria are correlated because genetic information has been transferred from AT-rich chromosomes to GC-rich chromosomes during evolution. If most bacteria could acquire DNAs with GC content higher than that in their own chromosome in addition to the lower GC content DNAs, then the flow of DNA fragments would not be biased and the genome size and GC content would not show a correlation. The results of previous studies indicate that bacteria contain a system (or systems) to generate and maintain GC content differences in the chromosomal DNA (e.g., Lawrence and Ochman, 1997). In order to maintain GC content, bacteria should estimate the DNA base composition of the transferred DNA fragments.

**Figure 1:**
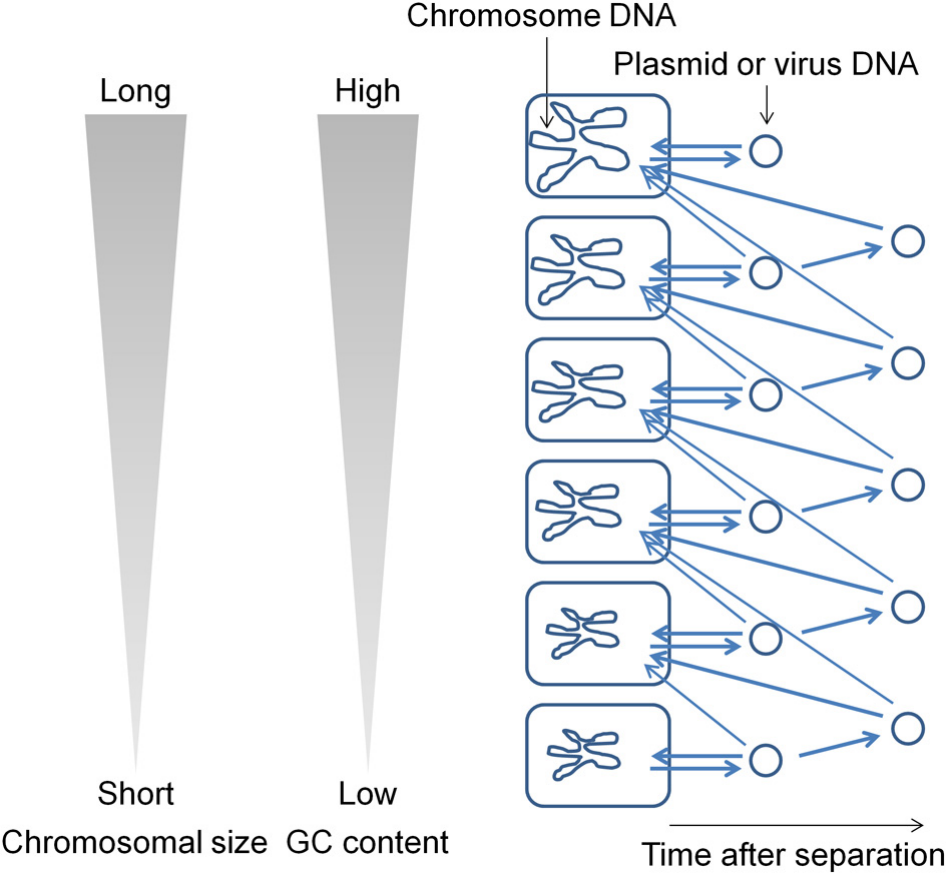
**Flow of DNA fragments in bacteria**.

## References

[B1] Ali S. S. Xia B. Liu J. Navarre W. W. (2012). Silencing of foreign DNA in bacteria. Curr. Opin. Microbiol. 15, 175–181 10.1016/j.mib.2011.12.014 22265250

[B2] Bentley S. D. Parkhill J. (2004). Comparative genomic structure of prokaryotes. Ann. Rev. Genet. 38, 771–792 10.1146/annurev.genet.38.072902.094318 15568993

[B3] Castang S. McManus H. R. Turner K. H. Dove S. L. (2008). H-NS family members function coordinately in an opportunistic pathogen. Proc. Natl. Acad. Sci. U.S.A. 105, 18947–18952 10.1073/pnas.0808215105 19028873 PMC2596223

[B4] Davison J. (1999). Genetic exchange between bacteria in the environment. Plasmid 42, 73–91 10.1006/plas.1999.1421 10489325

[B5] Gordon B. R. G. Li Y. Cote A. Weirauch M. T. Ding P. Hughes T. R. (2011). Structural basis for recognition of AT-rich DNA by unrelated exnogeneic silencing proteins. Proc. Natl. Acad. Sci. U.S.A. 108, 10690–10695 10.1073/pnas.1102544108 21673140 PMC3127928

[B6] Guo F. B. Lin H. Huang J. (2009). A plot of G + C content against sequence length of 640 bacterial chromosomes shows the points are widely scattered in the upper triangular area. Chromosome Res. 17, 359–364 10.1007/s10577-009-9024-3 19283496

[B7] Harrison E. Brockhurst M. A. (2012). Plasmid-mediated horizontal gene transfer is a coevolutionary process. Trends Microbiol. 20, 262–267 10.1016/j.tim.2012.04.003 22564249

[B8] Hershberg R. Petrov D. A. (2010). Evidence that mutation is universally biased towards AT in bacteria. PLoS Genet. 6:e1001115 10.1371/journal.pgen.1001115 20838599 PMC2936535

[B9] Hildebrand F. Meyer A. Eyre-Walker A. (2010). Evidence of selection upon genomic GC-content in bacteria. PLoS Genet. 6:e1001107 10.1371/journal.pgen.1001107 20838593 PMC2936529

[B10] Lawrence J. G. Ochman H. (1997). Amelioration of bacterial genomes: rates of change and exchange. J. Mol. Evol. 44, 383–397 10.1007/PL00006158 9089078

[B11] Lind P. A. Andersson D. I. (2008). Whole-genome mutational biases in bacteria. Proc. Natl. Acad. Sci. U.S.A. 105, 17878–17883 10.1073/pnas.0804445105 19001264 PMC2584707

[B12] Lucchini S. Rowley G. Goldberg M. D. Hurd D. Harrison M. Hinton J. C. (2006). H-NS mediates the silencing of laterally acquired genes in bacteria. PLoS Pathog. 2:e81 10.1371/journal.ppat.0020081 16933988 PMC1550270

[B13] McCutcheon J. P. Moran N. A. (2010). Functional convergence in reduced genomes of bacterial symbionts spanning 200 My of evolution. Genome Biol. Evol. 2, 708–718 10.1093/gbe/evq055 20829280 PMC2953269

[B14] McCutcheon J. P. Moran N. A. (2012). Extreme genome reduction in symbiotic bacteria. Nat. Rev. Microbiol. 10, 13–26 10.1038/nrmicro2670 22064560

[B15] Mira A. Klasson L. Andersson S. G. (2002). Microbial genome evolution: sources of variability. Curr. Opin. Microbiol. 5, 506–512 10.1016/S1369-5274(02)00358-2 12354559

[B16] Mira A. Ochman H. Moran N. A. (2001). Deletional bias and the evolution of bacterial genomes. Trends Genet. 17, 589–596 10.1016/S0168-9525(01)02447-7 11585665

[B17] Mitchell D. (2007). GC content and genome length in Chargaff compliant genomes. Biochem. Biophys. Res. Commun. 353, 207–210 10.1016/j.bbrc.2006.12.008 17173863

[B18] Moran N. A. (2002). Microbial minimalism: genome reduction in bacterial pathogens. Cell 108, 583–586 10.1016/S0092-8674(02)00665-7 11893328

[B19] Moran N. A. McCutcheon J. P. Nakabachi A. (2008). Genomics and evolution of heritable bacterial symbionts. Annu. Rev. Genet. 42, 165–190 10.1146/annurev.genet.41.110306.130119 18983256

[B20] Musto H. Naya H. Zavala A. Romero H. Alvarez-Valín F. Bernardi G. (2006). Genomic GC level, optimal growth temperature, and genome size in prokaryotes. Biochem. Biophys. Res. Commun. 347, 1–3 10.1016/j.bbrc.2006.12.008 16815305

[B21] Navarre W. W. Porwollik S. Wang Y. McClelland M. Rosen H. Libby S. J. (2006). Selective silencing of foreign DNA with low GC content by the H-NS protein in *Salmonella*. Science 313, 236–238 10.1126/science.1128794 16763111

[B22] Nilsson A. I. Koskiniemi S. Eriksson S. Kugelberg E. Hinton J. C. D. Andersson D. I. (2005). Bacterial genome size reduction by experimental evolution. Proc. Natl. Acad. Sci. U.S.A. 102, 12112–12116 10.1073/pnas.0503654102 16099836 PMC1189319

[B23] Nishida H. (2012a). Comparative analyses of base composition, DNA sizes, and dinucleotide frequency profiles in archaeal and bacterial chromosomes and plasmids. Int. J. Evol. Biol. 2012, 342482 10.1155/2012/342482 22536540 PMC3321278

[B24] Nishida H. (2012b). Nucleosome positioning. ISRN Mol. Biol. 2012, 245706 10.5402/2012/245706 PMC4890889 27335664

[B25] Nishida H. (2013). Genome DNA sequence variation, evolution, and function in bacteria and archaea. Curr. Issues Mol. Biol. 15, 19–24 22772895

[B26] Rocha E. P. Danchin A. (2002). Base composition bias might result from competition for metabolic resources. Trends Genet. 18, 291–294 10.1016/S0168-9525(02)02690-2 12044357

[B27] Rocha E. P. Feil E. J. (2010). Mutational patterns cannot explain genome composition: are there any neutral sites in the genomes of bacteria? PLoS Genet. 6:e1001104 10.1371/journal.pgen.1001104 20838590 PMC2936526

[B28] Smits W. K. Grossman A. D. (2010). The transcriptional regulator Rok binds A+T-rich DNA and is involved in repression of a mobile genetic element in *Bacillus subtilis*. PLoS Genet. 6:e1001207 10.1371/journal.pgen.1001207 21085634 PMC2978689

[B29] Song H. Hwang J. Yi H. Ulrich R. L. Yu Y. Nierman W. C. (2010). The early stage of bacterial genome-reductive evolution in the host. PLoS Pathog. 6:e1000922 10.1371/journal.ppat.1000922 20523904 PMC2877748

[B30] Sørensen S. J. Bailey M. Hansen L. H. Kroer N. Wuertz S. (2005). Studying plasmid horizontal transfer *in situ*: a critical review. Nat. Rev. Microbiol. 3, 700–710 10.1038/nrmicro1232 16138098

[B31] Sueoka N. (1988). Directional mutation pressure and neutral molecular evolution. Proc. Natl. Acad. Sci. U.S.A. 85, 2653–2657 3357886 10.1073/pnas.85.8.2653 PMC280056

[B32] Suzuki H. Sota M. Brown C. J. Top E. M. (2008). Using Mahalanobis distance to compare genomic signatures between bacterial plasmids and chromosomes. Nucleic Acids Res. 36, e147 10.1093/nar/gkn753 18953039 PMC2602791

[B33] Takeda T. Yun C.-S. Shintani M. Yamane H. Nojiri H. (2011). Distribution of genes encoding nucleoid-associated protein homologs in plasmids. Int. J. Evol. Biol. 2011, 685015 10.4061/2011/685015 21350637 PMC3042613

[B34] Thomas S. H. Wagner R. D. Arakaki A. K. Skolnick J. Kirby J. R. Shimkets L. J. (2008). The mosaic genome of *Anaeromyxobacter dehalogenans* strain 2CP-C suggests an aerobic common ancestor to the delta-Proteobacteria. PLoS ONE 3:e2103 10.1371/journal.pone.0002103 18461135 PMC2330069

[B35a] Wang W. Li G.-W. Chen C. Xie X. S. Zhuang X. (2011). Chromosome organization by a nucleoid-associated protein in live bacteria. Science 333, 1445–1449 10.1126/science.1204697 21903814 PMC3329943

[B35] Wu H. Zhang Z. Hu S. Yu J. (2012). On the molecular mechanism of GC content variation among eubacterial genomes. Biol. Direct 7, 2 10.1186/1745-6150-7-2 22230424 PMC3274465

[B36] Yun C. S. Suzuki C. Naito K. Takeda T. Takahashi Y. Sai F. (2010). Pmr, a histone-like protein H1 (H-NS) family protein encoded by the lncP-7 plasmid pCAR1, is a key global regulator that alters host function. J. Bacteriol. 192, 4720–4731 10.1128/JB.00591-10 20639326 PMC2937398

[B37] Zhao X. Zhang Z. Yan J. Yu J. (2007). GC content variability of eubacteria is governed by the pol III alpha subunit. Biochem. Biophys. Res. Commun. 356, 20–25 10.1016/j.bbrc.2007.02.109 17336933

